# A Model-Based Public-Payer Investment Appraisal of a National Home Hemodialysis Program in Greece: A Net Present Value Analysis

**DOI:** 10.3390/jmahp14030051

**Published:** 2026-08-27

**Authors:** Vasileios Zavvos, John Fanourgiakis, Michael A. Talias, Christos Iatrou, Christos Ntais

**Affiliations:** 1 Healthcare Management Program, School of Social Sciences, Hellenic Open University, 26335 Patras, Greece; 2 Centre for Nephrology and Dialysis Unit, HYGEIA Hospital, 15123 Athens, Greece; 3 Department of Management Science and Technology, Hellenic Mediterranean University, 72100 Agios Nikolaos, Greece; jfanourgiakis@hmu.gr; 4 Healthcare Management Program, School of Economics & Management, Open University of Cyprus, Nicosia 2220, Cyprus; michael.talias@ouc.ac.cy

**Keywords:** home hemodialysis, net present value, budget impact, public payer, kidney replacement therapy, dialysis costs, Greece

## Abstract

Background: In-center hemodialysis is the dominant kidney replacement therapy modality in Greece and generates substantial recurring expenditure for the public payer. Home hemodialysis is not currently implemented at national scale, but it may reduce long-term public expenditure if early investment in training capacity, equipment and home support is recovered over time. Objective: To evaluate, from the Greek public-payer perspective, the discounted budget impact and net present value (NPV) of implementing a national home hemodialysis program for 300 patients. Methods: We developed a deterministic investment-appraisal model comparing gradual implementation of home hemodialysis with continued in-center hemodialysis for the same projected cohort over 10 years. The in-center comparator was informed by a 2022 Greek patient-level micro-costing study. Home hemodialysis expenditure was constructed from explicit patient-flow equations, resource quantities, unit costs, capital purchases and hospital-tariff offsets. Annual incremental savings were discounted at 3% in the base case. Alternative discount rates, deterministic one-way sensitivity analyses and program-scale scenarios were examined. Fiscal benefit–cost ratio (BCR) and return on investment (ROI) were also calculated. Results: Undiscounted 10-year public expenditure was EUR 69,681,804 for home hemodialysis and EUR 86,700,267 for continued in-center hemodialysis, yielding savings of EUR 17,018,463. During the first 5 years, the program required EUR 1,724,012 in additional expenditure. At a 3% discount rate, NPV was EUR 12,696,564, the fiscal BCR was 2.89, fiscal ROI was 189.3% and discounted payback occurred during year 6. NPV remained positive at 5% (EUR 10,391,382) and across all tested one-way scenarios (range EUR 706,179 to EUR 24,686,948). Conclusions: The modeled national home hemodialysis program generated a positive 10-year public-payer NPV under the base-case and tested sensitivity assumptions. A positive NPV is not, however, a formal Greek health-system decision rule and does not capture health outcomes, patient and family costs, or equity. The findings support staged pilot implementation and prospective collection of Greek real-world data before wider rollout.

## 1. Introduction

Kidney failure requiring kidney replacement therapy is a high-cost chronic condition for health systems. Although the treated population is relatively small, dialysis generates recurring expenditure for dialysis sessions, consumables, medicines, laboratory monitoring, patient transportation, hospitalization and dialysis-unit infrastructure. In Greece, in-center hemodialysis (ICHD) remains the predominant modality, while home hemodialysis (HHD) has not developed as a routine national treatment pathway. A recent Greek public-payer micro-costing study quantified the direct costs of dialysis therapies and provided contemporary estimates that can be used as comparator inputs for health economic modeling [1]. European and international renal registries further demonstrate that dialysis modality mix, access to home therapies and long-term affordability of kidney replacement therapy remain central policy issues for health systems [2,3].

HHD has attracted renewed international interest because it shifts part of the treatment pathway from a facility-based model to the patient’s home. From a patient perspective, home treatment may increase autonomy, reduce travel burden and permit more flexible scheduling [4,5,6,7,8]. However, HHD is not merely a change of treatment location; implementation requires coordinated clinical, technical, and logistical support [5,6,7,8,9,10,11,12,13,14].

Patients who can start HHD are unlikely to be a random subset of the ICHD population. Conventional self-care HHD generally requires clinical stability, a usable vascular access, cognitive and physical capacity to perform treatment or reliable care-partner assistance, an adequate home environment, and willingness to assume treatment tasks [5,6,7,8,12,14]. Early HHD cohorts may therefore be younger, less frail or more able to self-manage than the average ICHD population. Geography can operate in both directions: patients living far from dialysis facilities may gain most from reduced travel, whereas remote regions may face greater challenges in home installation, consumable delivery, technical response and emergency backup. These differences affect both selection and costs and limit direct transfer of an unadjusted average ICHD cost to every potential HHD participant.

The economic attractiveness of HHD is therefore context-specific. Systematic reviews and economic evaluations have generally found that HHD can be less costly than facility hemodialysis in settings where training, equipment, technical support and follow-up are organized efficiently [4,15,16,17,18,19,20,21,22,23]. Nevertheless, the magnitude and timing of savings depend on reimbursement design, local staffing costs, procurement prices, patient recruitment, patient retention on HHD (technique survival) and the ability of the program to spread fixed costs over a sufficiently large active patient population. These determinants are particularly relevant in Greece, where the first national HHD program would have to create new capacity before mature savings could be realized.

Implementation guidance for HHD emphasizes that program development requires more than machine procurement. Successful expansion depends on modality education, shared decision-making, trained dialysis nurses, clinical governance, emergency procedures, home water and electrical safety, delivery of consumables, waste management and a reimbursement mechanism that does not penalize hospitals for moving care out of the facility [5,6,7,8,9,10,11,12,13,14]. These elements generate initial investment costs that are concentrated in the early years of implementation. Potential savings, in contrast, accrue gradually as patients complete training and remain on home therapy.

This timing structure makes the Greek case suitable for investment appraisal. A cross-sectional comparison of annual per-patient costs would not answer whether a payer should finance a national program, because the payer must first fund training infrastructure, dedicated personnel, home adaptations, machines, water-treatment systems and logistics. The relevant fiscal question is whether these early costs are recovered by later reductions in public expenditure. Net present value (NPV) analysis places early outlays and later savings on a common present-value basis and is widely used in discounted cash-flow and public-investment appraisal, commonly alongside benefit–cost ratio (BCR) and return on investment (ROI) indicators [24,25,26,27]. In the present health-system application, NPV addresses an investment and affordability question rather than cost-effectiveness. No formal Greek health-system NPV threshold for dialysis service redesign is specified in the sources used for this analysis; therefore, a positive NPV should not be interpreted as an automatic priority over competing interventions.

The study hypothesis was that, despite front-loaded implementation expenditure, gradual establishment of a 300-patient national HHD program in Greece would generate a positive 10-year public-payer NPV compared with continued ICHD for the same projected cohort, provided that the modeled recruitment and retention pathway was achieved. The primary objective was to estimate this 10-year NPV. Secondary objectives were to quantify the undiscounted budget impact, present value of expenditures at alternative discount rates, discounted payback year, maximum discounted funding requirement, fiscal BCR and ROI, and to assess robustness through deterministic one-way sensitivity and program-scale scenarios.

## 2. Materials and Methods

### 2.1. Study Design and Perspective

This was a deterministic, model-based public-payer investment appraisal and budget-impact analysis. The analysis compared two strategies over a 10-year horizon: continued ICHD versus gradual implementation of a national HHD program. The economic question was not whether HHD improves survival or quality-adjusted life-years, but whether the projected public expenditure avoided by shifting a defined cohort from ICHD to HHD exceeds the expenditure required to establish and operate the program.

The analysis adopted the perspective of the Greek public payer and publicly financed hospital system. Included costs were those expected to be borne by the National Organization for the Provision of Health Services (EOPYY), public hospitals or the publicly financed health budget under the modeled implementation pathway. The model did not monetize patient time, caregiver time, productivity, informal care, out-of-pocket costs or home-space constraints. The results should therefore be interpreted as a fiscal investment appraisal rather than a societal economic evaluation.

The analysis was prepared with reference to the CHEERS 2022 reporting framework where applicable, the ISPOR principles for budget impact analysis and general health economic evaluation methods [24,25,26,28,29,30]. Because budget-impact analysis and investment appraisal answer different questions from cost-utility analysis, the emphasis was placed on transparent cost streams, timing of expenditures, comparator definition, discounting and sensitivity analysis rather than on incremental cost-effectiveness ratios.

### 2.2. Data Sources and Model Inputs

The ICHD and peritoneal dialysis (PD) comparator inputs were obtained from a recent public-payer micro-costing study, which used calendar-year 2022 patient-level data from five Greek hemodialysis units (two public hospital units, two private hospital units and one private chronic hemodialysis center) and one public-hospital PD unit [1]. Eligible adults received the same dialysis modality continuously throughout 2022. The final analytic sample included 359 patients: 337 receiving hemodialysis and 22 receiving PD. Annual costs were measured from the public-payer/public-budget perspective and included treatment and consumables, patient allowances and transport reimbursement, dialysis-related medicines, laboratory and imaging tests, hospitalizations and unit operating costs where applicable. Public-hospital operating costs were included through accounting allocations, whereas private-provider operating costs were not added separately because they are financed within the reimbursed tariff and are not separately reimbursed by EOPYY; the source estimates therefore represent recorded public expenditure rather than full economic production cost.

The base-case ICHD input, EUR 35,737.95 per patient-year, was the source study’s national subtype-weighted mean annual HD cost, calculated from the mean costs of conventional hemodialysis (HD) (EUR 33,085.43) and hemodiafiltration (HDF) (EUR 41,093.26) using 2022 national subtype counts. The contextual PD input, EUR 63,557.10 per patient-year, was similarly weighted across continuous ambulatory peritoneal dialysis (CAPD) and automated peritoneal dialysis (APD). The micro-costing hemodialysis cohort had a median age of 70 years and 75.1% received treatment in private-sector units, but the study did not define HHD eligibility or report frailty, clinical severity, geographic region, distance to a dialysis unit or home-treatment accessibility [1]. The subtype-weighted HD estimate was therefore used as an aggregate public-expenditure comparator rather than a matched estimate for a typical HHD-eligible patient. To examine cost and case-mix uncertainty, one-way sensitivity analysis used the conventional HD and HDF mean costs as lower and upper comparator values.

All HHD-specific annual costs were constructed within the present model using explicit statutory-tariff inputs, procurement and resource-use estimates, published HHD implementation literature and expert-informed assumptions where Greek data were unavailable. Expert-informed assumptions were restricted to implementation-specific parameters in a country without an established national HHD pathway, including feasible training throughput, staffing, home-installation logistics, equipment procurement and consumable delivery. These assumptions define a transparent base-case policy scenario rather than observed Greek HHD practice. All monetary inputs were valued in 2022 euros and the analysis was conducted in constant-price (real) terms. Unit costs and annual cash flows were not escalated for general inflation. The inputs are reported in Table 1.

The primary comparator was the national subtype-weighted mean annual public-payer/public-budget cost of continued ICHD, EUR 35,737.95 per patient-year, obtained from the Greek micro-costing study [1]. A secondary contextual benchmark used the corresponding national subtype-weighted mean PD cost, EUR 63,557.10 per patient-year [1]. Because aggregate expenditure is driven by mean rather than median cost, the mean-based national HD estimate was used in the base case. PD was not considered clinically interchangeable with HHD for all patients and was not used to rank HHD against a separately modeled PD investment program. Instead, Appendix A Table A1 reports PD and ICHD public-payer cost streams for the same projected patient equivalents as a transparent budget benchmark.

### 2.3. Home Hemodialysis Program Scenario

The HHD scenario represented a national program reaching a policy cap of 300 active patients. Ten public hospitals were assumed to participate, each training 15 patients annually, for a national throughput of 150 patients per year. Training required 40 hemodialysis sessions per patient. Each hospital was allocated two training machines and two dedicated nurses. HHD was modeled as conventional treatment delivered three times weekly, corresponding to 156 sessions per patient-year.

Patient flow was constructed explicitly. Let *E_t_* denote the active HHD population at the end of year *t*, *R* the annual training throughput (150 patients) and *d* the annual attrition/non-retention rate (0.30). During scale-up, *E_t_* = (*E_t_*_−1_ + *R*) × (1 − *d*), with *E*_0_ = 0. The average active patient equivalent during year *t* was *A_t_* = (*E_t−_*_1_ + *E_t_*)/2. Using rounding annual patient equivalents to whole patients generated *A_t_* values of 53, 142, 204, 248, and 279 in years 1–5. From year 6 onward, the HHD program scenario capped *A_t_* at 300.

For one-off home-preparation and household-equipment expenditure, annual successful home installations were calculated as *H_t_* = *R* × (1 − *d*) = 105. The model retained 105 installations annually, including after the 300-patient cap, as a conservative replacement and within-year turnover assumption. The annual number of HHD sessions (*S_t_*) was *S_t_* = 156 × *A_t_*. These equations, rather than externally supplied annual totals, generated the patient-flow and utilization inputs used in the cost model.

### 2.4. Home Hemodialysis Cost Construction

HHD program cost inputs were estimated using Greek dialysis micro-costing data [1], statutory reimbursement schedules, market procurement prices, utility-cost estimates, published HHD implementation literature and expert-informed assumptions. Table 2 reports every HHD-specific unit-cost input used in the base case. The annual public cost was divided into (i) direct patient-level public expenditure and (ii) the additional net operating cost of the hospital HHD program.

Gross annual program expenditure (*O_t_*) was constructed as the sum of the training seminar (*TrS*), staffing, home visits, facilities/administration, home adaptations, equipment purchases, technical support, household equipment, utilities, water testing, waste management and consumable delivery:

*O_t_* = *TrS* + 650,000 + 150*A_t_* + 240,000 + 2500*H_t_* + 35,000*M_t_* + 2400*K_t_* + 2100*H_t_* + (750 + 300 + 500 + 1000)*A_t_*

The public payer reimburses the participating public hospital for each HHD session. Annual session-tariff revenue (*R_t_*) to the hospital was therefore *R_t_* = 105 × 156 × *A_t_* = 16,380*A_t_*. The additional public-hospital program burden (*G_t_*) was *G_t_* = *O_t_* − *R_t_*. A negative *G_t_* indicates that session-tariff revenue exceeds the modeled program operating expenditure in that year; it does not imply a negative total public-payer cost.

Direct patient-level public expenditure (*D_t_*) was calculated independently as follows:

*D_t_* = *A_t_* × (16,380 + 3052.30 + 4544 + 3300 + 580 + 1850) = 29,706.30*A_t_*

Total annual HHD public expenditure (*C_HHD,t_*) was then calculated as

C_HHD,t_ = D_t_ + G_t_

The session tariff paid by EOPYY was included once as public-payer expenditure. Because the same payment constitutes revenue for the public hospital, only the portion of gross hospital program costs not covered by the tariff was added to the consolidated public-payer cost.

### 2.5. Net Present Value Calculation

The primary outcome was the NPV of HHD relative to continued ICHD. The annual in-center comparator was the published national subtype-weighted HD estimate from Zavvos et al. [1] and was applied to the same annual patient equivalents: *C_ICHD,t_* = EUR 35,737.95 × *A_t_*. Contextual PD expenditure was *C_PD,t_* = EUR 63,557.10 × *A_t_*. The NPV was calculated as the discounted difference between annual ICHD expenditure and constructed annual HHD expenditure:

$$NPV=\sum_{t=1}^{T}\frac{C_{ICHD,t}-C_{HHD,t}}{{\left(1+r\right)}^{t}}$$

In this expression, NPV denotes the net present value of the HHD program relative to continued ICHD; *t* denotes the model year; *T* is the 10-year horizon; *C_ICHD,t_* is the ICHD expenditure in year *t*; *C_HHD,t_* is the constructed HHD expenditure in year *t*; *r* is the annual discount rate; and (1 *+ r*)*^t^* converts each future expenditure difference to present value.

A positive NPV indicates that the discounted value of avoided ICHD expenditure exceeds the discounted cost of implementing and operating the HHD program within the selected horizon. A negative NPV would indicate that the program does not recover its investment within that horizon. This sign rule is an accounting interpretation, not a formal Greek health-system adoption threshold. Expenditures were assumed to occur at the end of each year. No separate initial investment term was subtracted outside the annual cost streams because equipment, training, home-installation and other start-up costs were already incorporated into annual HHD expenditure.

In addition to the NPV, we calculated undiscounted cumulative expenditure, present value of HHD and comparator expenditure at alternative discount rates, the discounted payback year and the maximum cumulative discounted funding requirement. The payback year was the first year in which cumulative discounted incremental savings became positive. The maximum funding requirement was the largest negative value of cumulative discounted incremental savings before payback.

To provide indicators that are less dependent on program size than absolute NPV, the present value of incremental investment was calculated as the discounted sum of years in which HHD expenditure exceeded ICHD expenditure, and the present value of gross savings as the discounted sum of years in which ICHD expenditure exceeded HHD expenditure. Fiscal BCR was defined as present value of gross savings divided by present value of incremental investment. Fiscal ROI was defined as NPV divided by present value of incremental investment, equivalent to BCR minus 1. These indicators include only public-payer cash flows; they are not a societal cost-benefit analysis and do not monetize QALYs, survival, patient time or caregiver effects.

### 2.6. Sensitivity and Scenario Analyses

The base-case discount rate was 3%, with sensitivity analyses at 0%, 1.5% and 5%; all rates were applied to cash flows expressed in constant 2022 euros. No general inflation was modeled, so the non-zero rates were interpreted as real discount rates rather than adjustments for nominal price growth. Because HHD requires early start-up expenditure but generates savings later, these rates assessed whether the program remained budget-favorable under different weights assigned to future savings. A 0% rate reproduces the undiscounted budget impact, whereas higher rates reduce the present value of later savings [26].

Deterministic one-way sensitivity analyses were conducted at the 3% discount rate. The tested values were the following: annual ICHD comparator cost EUR 33,085.43 and EUR 41,093.26; attrition/non-retention 20% and 40%; national training throughput 100 and 200 patients per year; dedicated staff cost ±20%; machine/water-system acquisition cost ±20%; technical-support cost ±20%; home-preparation and household-equipment cost ±20%; direct patient-level HHD public cost ±20%; and staff-training seminar cost ±50%. All other parameters were held at base-case values. Patient flows and successful home installations were recalculated in the attrition and throughput analyses.

Program-scale scenarios examined a 240-patient program with annual throughput of 120 and a 360-patient program with annual throughput of 180. Machine and water-system capacity and procurement were adjusted proportionally to the target population. The ten-site network and base-case dedicated staffing and facilities budgets were held constant to isolate the effect of spreading fixed program costs over smaller or larger active populations.

The PD analysis was secondary and contextual. It applied the published national subtype-weighted mean PD cost to the same projected patient equivalents and compared the resulting PD public-payer stream with continued ICHD. It did not assume that HHD and PD are clinically interchangeable, and it did not calculate an HHD-versus-PD investment NPV because a distinct PD implementation pathway, start-up investment and patient-selection model were not specified. Year-by-year PD and ICHD cost streams are reported in Appendix A Table A1.

## 3. Results

### 3.1. Program Expenditure and Undiscounted Budget Impact

The complete HHD unit-cost construction is reported in Table 3.

The undiscounted 10-year HHD expenditure was consequently EUR 69,681,804.

The formulas and unit inputs in Table 2 and Table 3 generated the annual HHD expenditure used in the investment appraisal. The comparison with continued ICHD is presented in Table 4. HHD was more expensive during the first 2 years because equipment and training expenditure was incurred before the active cohort reached scale. The incremental budget impact was EUR −4,553,283 in year 1 and EUR −2,423,946 in year 2.

The annual budget impact became positive in year 3, when constructed HHD expenditure was EUR 6,705,365 compared with EUR 7,290,542 for continued ICHD, generating annual savings of EUR 585,177. From year 6 onward, with 300 active patient equivalents, annual HHD expenditure was EUR 6,972,890 compared with EUR 10,721,385 for continued ICHD, generating annual undiscounted savings of EUR 3,748,495.

Over the 10-year horizon, undiscounted expenditure was EUR 69,681,804 for HHD and EUR 86,700,267 for continued ICHD. Undiscounted savings were therefore EUR 17,018,463. During the first 5 years, cumulative HHD expenditure exceeded the comparator by EUR 1,724,012, demonstrating that the program should be interpreted as an investment with medium-term payback rather than an immediate cost-saving intervention.

### 3.2. Net Present Value and Payback

At the 3% base-case discount rate, the present value of HHD expenditure was EUR 59,428,165 and the present value of continued ICHD expenditure was EUR 72,124,728 (Table 5). The resulting NPV was EUR 12,696,564. The present value of incremental investment was EUR 6,705,464 and the present value of gross savings was EUR 19,402,028, producing a fiscal BCR of 2.89 and fiscal ROI of 189.3%. Cumulative discounted savings remained negative through year 5 and became positive during year 6.

The NPV remained positive in all tested discount-rate scenarios. With no discounting, NPV was EUR 17,018,463. At 1.5%, NPV was EUR 14,712,435; at 5%, it remained positive at EUR 10,391,382. The persistence of positive NPV at 5% indicates that the result is not dependent solely on distant savings.

### 3.3. Deterministic Sensitivity and Program-Scale Analyses

NPV remained positive in all deterministic one-way analyses (Appendix A Table A2), ranging from EUR 706,179 when direct patient-level HHD public cost was increased by 20% to EUR 24,686,948 when that cost was reduced by 20%. Using the conventional hemodialysis mean as the ICHD comparator produced an NPV of EUR 7,343,367; 40% attrition/non-retention produced EUR 3,126,623; and training throughput of 100 patients per year produced EUR 1,927,361. Across the tested one-way scenarios, discounted payback occurred between years 5 and 10. The results therefore remained budget-favorable, but the narrow margin in the high HHD patient-cost scenario demonstrates material parameter uncertainty.

In the fixed-network scale scenarios (Appendix A Table A3), the 240-patient program generated an NPV of EUR 8,596,628, a fiscal BCR of 2.50 and payback in year 7. The 360-patient program generated an NPV of EUR 16,741,778, a fiscal BCR of 3.17 and payback in year 6. These scenarios illustrate that absolute NPV increases with program size and that spreading fixed staffing and facilities costs over more active patients improves the fiscal return indicators.

### 3.4. Contextual Peritoneal Dialysis Budget Benchmark

At the 3% discount rate, the present value of PD public-payer expenditure for the same projected patient equivalents was EUR 128,268,089, compared with EUR 72,124,728 for continued ICHD. The discounted incremental PD expenditure relative to ICHD was therefore EUR 56,143,361. Appendix A Table A1 presents the annual PD and ICHD streams. This is a contextual budget difference rather than an investment NPV and should not be interpreted as evidence that PD and HHD, or PD and ICHD, are clinically interchangeable for individual patients.

## 4. Discussion

This model-based economic evaluation indicates that a national HHD program in Greece can generate a positive public-payer NPV under the modeled assumptions. It is not cost-saving immediately: the first 2 years are dominated by start-up and scale-up costs, and cumulative expenditure remains higher than the in-center comparator during the first 5 years. Once the active cohort approaches the 300-patient cap, annual savings accumulate and the 10-year NPV becomes positive. HHD should therefore be evaluated as a medium-term investment in service capacity rather than as a short-term cost-cutting intervention.

The timing of costs explains the result. ICHD is a mature recurring expenditure: each year of treatment generates session payments, materials, medicines, monitoring, transportation and hospital-related costs. An HHD program, by contrast, requires early expenditure on training infrastructure, dedicated staff, patient education, machines, water-treatment systems and home adaptation. These costs are incurred before the program reaches steady-state size. If recruitment is successful and attrition is manageable, the fixed and start-up costs are spread over a growing active cohort and the program begins to generate annual savings.

The positive NPV at 3% and 5% is informative but not sufficient for a national adoption decision. NPV is an absolute monetary measure and may favor larger projects; the scale scenarios show that a larger program produces a larger NPV. Fiscal BCR and ROI provide complementary scale-normalized indicators, while the maximum funding requirement and payback year describe affordability. Nevertheless, no formal Greek health-system NPV, BCR or ROI threshold for dialysis service redesign was identified in the methodological sources used here. A decision maker facing a constrained budget would still need to compare the required early funding, opportunity costs, health outcomes, equity effects, implementation feasibility and risks of HHD with those of competing investments. The present results therefore support the fiscal case for further implementation testing, not an automatic national funding decision [24,25,26,27,32].

These findings are consistent with international economic studies showing that HHD can be economically attractive but is sensitive to local implementation conditions [4,15,16,17,18,19,20,21,22,23]. Prior studies have emphasized training, staffing, equipment, patient retention and program scale [9,10,11,12,13,21,22]. The new one-way analyses reach the same conclusion. NPV remained positive across the tested ranges, but it fell to EUR 706,179 when direct patient-level HHD public cost was increased by 20%, to EUR 1,927,361 with annual training throughput of 100 patients and to EUR 3,126,623 with 40% attrition/non-retention. The investment case is therefore conditional on disciplined procurement, sufficient recruitment, technique retention and control of recurring patient-level costs.

## 5. Policy Implementation, Financing and Research Priorities

A sustainable Greek pathway would be staged. An initial pilot in a small number of experienced nephrology centers should establish standardized eligibility, training, home assessment, technical support, emergency backup and data collection. Expansion to regional hubs and then to the modeled ten-site network should occur only after predefined criteria are met for recruitment, training completion, technique survival, safety, technical response times, supply reliability, staff workload and budget performance. This approach allows capacity and affordability to be tested before irreversible national commitments are made.

Financing should combine ring-fenced public start-up funding with a reimbursement design that pays for the complete HHD pathway rather than only dialysis sessions. A bundled or add-on payment could explicitly cover patient education and training, home assessment and adaptation, home visits, remote monitoring, technical support, consumable delivery and backup care, while allowing hospitals to retain an appropriate share of savings from reduced facility treatment. Public-private contracts may be useful for equipment procurement, maintenance and logistics, but eligibility rules, quality standards, clinical accountability and data governance should remain publicly overseen. Sustainability should not depend on transferring electricity, water, storage, travel or caregiver costs to patients; such costs should be measured and, where material, offset through transparent allowances. International policy work supports aligned reimbursement and public-private supply arrangements, while recent evaluations show that financial incentives alone may have little effect and can disadvantage providers serving socially vulnerable populations unless equity safeguards are built in [33,34,35].

Avoiding underuse of available home capacity requires more than offering machines. All clinically appropriate patients should receive timely, balanced modality education and shared decision-making, supported by standardized rather than overly restrictive eligibility assessment, home visits, peer mentoring, retraining, respite and dependable temporary in-center backup. Assisted or care-partner-supported pathways could broaden access for selected patients who cannot perform every task independently. A mandatory home-first policy would be inappropriate. Uptake, refusal, dropout and outcomes should be monitored by region, age, sex, socioeconomic circumstances and distance from dialysis facilities so that remote access barriers and social-risk gradients are detected rather than reinforced [5,6,7,8,33,35,36].

The contextual PD analysis should be interpreted cautiously. PD and HHD are both home-based modalities, but they differ in clinical indications, patient burden, contraindications, training requirements and supply-chain structure. For that reason, the main analysis does not present an HHD-versus-PD NPV as if the two were competing investment projects. Appendix A Table A1 instead shows the PD and ICHD public-payer streams for the same projected patient equivalents. These values are useful for budget context but not for choosing an individual patient’s modality.

The research agenda should accompany implementation from the outset. A prospective national HHD registry should collect data on recruitment and eligibility, reasons for modality choice or refusal, training completion, technique survival, adverse events, vascular access outcomes, hospitalizations, mortality, health-related quality of life, patient-reported experience, staff workload, technical failures, home-installation and recurring program costs, patient out-of-pocket expenditure, time use and caregiver burden. These data would permit recalibration of the fiscal model, assignment of probability distributions for probabilistic sensitivity analysis, and development of a full cost-utility analysis using QALYs and survival. A societal cost-benefit analysis could additionally value patient, family and productivity effects. The fiscal BCR and ROI reported here are useful for the payer’s cash-flow question but are not substitutes for those broader analyses [24,25,28,32,37].

## 6. Strengths and Limitations

The main strength of this analysis is that it separates the investment phase from the mature operating phase and explicitly discounts future expenditure differences. This shows the payer when additional funding is required, when annual savings begin and when cumulative discounted savings become positive. The analysis also reports fiscal BCR, ROI, one-way sensitivity analyses and scale scenarios, and uses comparator costs from a recent Greek public-payer/public-budget micro-costing study rather than relying exclusively on international cost estimates [1].

A second strength is computational transparency. Patient flow, utilization, gross program expenditure, tariff revenue, net hospital program cost and total HHD expenditure can be reproduced from the equations and unit values in Table 1, Table 2 and Table 3. The comparator unit costs, by contrast, were intentionally taken directly from the recent Greek micro-costing study [1], avoiding unnecessary re-estimation of previously reported empirical results.

Several limitations should be emphasized. First, the HHD program has not yet been implemented nationally in Greece; training throughput, staffing, home-installation logistics, procurement and supply arrangements are therefore expert-informed assumptions rather than observed program data. Second, the micro-costing comparator represents the general treated hemodialysis population, not a matched HHD-eligible cohort. The source study did not report frailty, detailed clinical severity, geographic region, distance or home-accessibility measures. Selection and case-mix differences may therefore affect the true counterfactual cost. The source estimate is also a public-payer/public-budget measure rather than a full production-cost estimate because public-unit operating costs were visible while private-provider operating costs were not separately added. Source-based comparator ranges and other one-way analyses reduce, but do not eliminate, this uncertainty.

Third, deterministic one-way sensitivity analysis was performed, but no probabilistic sensitivity analysis was undertaken because empirical distributions and correlations were unavailable for several implementation parameters. One-way analysis cannot quantify joint uncertainty. Future work should assign distributions to recruitment, attrition, equipment, staffing, home-installation, consumables, hospitalization and maintenance costs and use Monte Carlo simulation [24,25,28,29,30]. Fourth, the model does not estimate QALYs, survival, quality of life or patient-reported outcomes and is not a full cost-utility or societal cost-benefit analysis. Fifth, recruitment and technique retention remain major uncertainties; the model conservatively retained 105 successful installations annually after the 300-patient cap, whereas a simple steady-state replacement calculation at 30% attrition would require 90. Sixth, patient and care-partner burdens, including storage, cannulation, machine set-up, water management, waste handling, time and anxiety about adverse events, were excluded [7,8,37].

## 7. Conclusions

The model results were consistent with the stated study hypothesis. Under the base-case assumptions, a national HHD program for 300 patients generated a 10-year public-payer NPV of EUR 12,696,564, a fiscal BCR of 2.89 and fiscal ROI of 189.3%, with discounted payback during year 6. NPV remained positive in all tested deterministic sensitivity analyses, although the margin narrowed materially under higher recurring HHD costs, slower training and greater attrition. Thus, within the modeled assumptions, discounted avoided ICHD expenditure exceeded HHD implementation and operating expenditure over 10 years. This finding is not a formal Greek health-system decision rule and should not be interpreted independently of clinical outcomes, equity, affordability, opportunity cost and implementation risk.

The results support staged pilot implementation rather than immediate nationwide rollout. A Greek pilot should include explicit start-up financing and reimbursement for the full home-care pathway and should prospectively collect patient-flow, clinical, quality-of-life, patient/family burden and cost data. These data would allow the fiscal model to be recalibrated, probabilistic uncertainty to be quantified and a full cost-utility and societal analysis to be undertaken before wider expansion.

## Figures and Tables

**Table 1:** Base-case model inputs and sources.

Model Parameter	Base-Case Value	Source/Calculation
Perspective	Greek public payer/publicly financed health system	Direct public expenditure only
Time horizon	10 years	Policy-relevant investment horizon
Discount rate	3% base case; 0%, 1.5% and 5% sensitivity analyses	Real rates applied to constant 2022-euro cash flows; health-economic and public-investment appraisal assumptions [24,25,26,27]
Comparator: ICHD	EUR 35,737.95 per patient-year	National subtype-weighted mean annual HD public-payer/public-budget cost from Zavvos et al. [1]; source-based sensitivity range EUR 33,085.43–EUR 41,093.26
Contextual comparator: PD	EUR 63,557.10 per patient-year	National subtype-weighted mean annual PD public-payer/public-budget cost from Zavvos et al. [1]; contextual benchmark only
Implementation sites	10 public hospitals	Base-case implementation assumption
Target active HHD population	300 patients	Policy implementation scenario
Training throughput	150 patients per year	15 patients per hospital per year
Annual attrition/non-retention	30%	Literature- and expert-informed assumption [5,6,7,8,14,31]
Successful home installations	105 per year	150 × (100% − 30%). Successful home installations were used to calculate one-off home-adaptation and household-equipment costs.
Training requirement	40 sessions per patient	HHD implementation literature [9,10,11,12,13]
Medical staff	5 physician full-time equivalents	10 hospitals × 1 physician × 50% of full-time hours
Dedicated nursing staff	2 nurses per hospital	10 hospitals × 2 nurses = 20 dedicated nurses nationally
HHD treatment schedule	3 sessions/week; 156 sessions/year	Conventional HHD schedule
Machine/water-system procurement	120, 110, 50 and 50 new sets in years 1–4	330 total sets (300-patient capacity + 10% reserve), acquired according to the annual schedule shown
Price basis	2022 euros; constant-price (real) analysis	No general inflation or nominal price escalation applied

HD: hemodialysis; HHD: home hemodialysis; ICHD: in-center hemodialysis; PD: peritoneal dialysis.

**Table 2:** HHD-specific unit-cost inputs and calculation rules.

Resource/Cost Item	Base-Case Input	Calculation Rule
Dialysis session reimbursement	EUR 105 per session	156 × *A_t_* × EUR 105 = EUR 16,380 × *A_t_* annually
Dialyzers, blood lines and needles	EUR 3052.30 per patient-year	EUR 3052.30 × *A_t_*
Patient allowances	EUR 4544 per patient-year	EUR 4544 × *A_t_*
Dialysis-related medicines	EUR 3300 per patient-year	EUR 3300 × *A_t_*
Laboratory tests	EUR 580 per patient-year	EUR 580 × *A_t_*
Hospitalizations	EUR 1850 per patient-year	EUR 1850 × *A_t_*
Staff training seminar	EUR 25,000 in years 1 and 3	One-week program-wide seminar
Medical and nursing staff	EUR 650,000 per year	20 dedicated nurses + 5 physician full-time equivalents
Home visits	EUR 150 per active patient-year	Four visits per patient-year; annual travel allowance EUR 150 × *A_t_*
Facilities and administration	EUR 240,000 per year	10 hospitals × EUR 23,223.65 = EUR 232,236; rounded model budget EUR 240,000
Home electrical/plumbing adaptation	EUR 2500 per successful installation	EUR 2500 × *H_t_*; *H_t_* = 105 annually
Dialysis machine	EUR 30,000 per machine	One machine per newly established HHD patient
Water-treatment system	EUR 5000 per system	Combined machine/system set = EUR 35,000
New machine/water-system sets *M_t_*	EUR 35,000 per set; 330 total sets = 300 target capacity + 10% reserve	EUR 35,000 × *M_t_* = EUR 35,000 × 120, 110, 50, 50 in years 1–4
Technical support *K_t_*	EUR 2400 per covered set-year; paid after two cost-free years	EUR 2400 × *K_t_* = EUR 2400 × 0, 0, 120, 230, 280, 330 in years 1–6 *; the 330 sets covered from year 6 onward generate a technical-support cost of 330 × EUR 2400 = EUR 792,000 annually
Household equipment	EUR 2100 per successful installation	EUR 2100 × *H_t_*; *H_t_* = 105 annually
Electricity and water	EUR 750 per active patient-year	(EUR 600 electricity + EUR 150 water) × *A_t_*
Water quality testing	EUR 300 per active patient-year	EUR 300 × *A_t_*
Infectious-waste management	EUR 500 per active patient-year	EUR 500 × *A_t_*
Consumable delivery	EUR 1000 per active patient-year	EUR 1000 × *A_t_*

* Year 6 values were applied to each of the years 6–10. *A_t_* denotes average active HHD patient equivalents in year *t*; *H_t_* denotes successful home installations; *M_t_* denotes newly purchased machine/water-system sets; *K_t_* denotes installed sets subject to paid technical support.

**Table 3:** Construction of annual HHD public expenditure (EUR).

Calculation Component	Year 1	Year 2	Year 3	Year 4	Year 5	Year 6 *
Gross program expenditure, *O_t_*	5,741,100	5,606,400	3,986,800	4,344,600	2,798,300	2,975,000
Session-tariff revenue, *R_t_*	868,140	2,325,960	3,341,520	4,062,240	4,570,020	4,914,000
Net program cost, *G_t_*	4,872,960	3,280,440	645,280	282,360	−1,771,720	−1,939,000
Direct patient-level public cost, *D_t_*	1,574,434	4,218,295	6,060,085	7,367,162	8,288,058	8,911,890
Total HHD public expenditure, *C_HHD,t_*	6,447,394	7,498,735	6,705,365	7,649,522	6,516,338	6,972,890

* Year 6 values were applied to each of the years 6–10. The non-monotonic pattern in gross program expenditure (*O_t_*) reflects the timing of capital purchases and technical support rather than instability in patient flow. *O_t_* decreases in year 3 because new machine/water-system purchases fall from 110 to 50 sets, more than offsetting the year-3 training seminar, the start of technical-support payments and the larger active cohort. It rises in year 4 because the active cohort grows and the number of sets under paid support increases from 120 to 230 while 50 additional sets are purchased. It decreases in year 5 because equipment acquisition ends and no seminar is scheduled, and it rises in year 6 because the active population reaches 300 and all 330 sets become subject to paid technical support. Year-6 expenditure then remains constant through year 10 under steady-state assumptions.

**Table 4:** Annual public-payer cost streams and discounted incremental savings at 3% (EUR).

Year	Patients	HHD Cost	ICHD Cost	Incremental Savings	Discounted Incremental Savings (3%)
1	53	6,447,394	1,894,111	−4,553,283	−4,420,663
2	142	7,498,735	5,074,789	−2,423,946	−2,284,801
3	204	6,705,365	7,290,542	585,177	535,519
4	248	7,649,522	8,863,012	1,213,489	1,078,169
5	279	6,516,338	9,970,888	3,454,550	2,979,925
6	300	6,972,890	10,721,385	3,748,495	3,139,306
7	300	6,972,890	10,721,385	3,748,495	3,047,869
8	300	6,972,890	10,721,385	3,748,495	2,959,097
9	300	6,972,890	10,721,385	3,748,495	2,872,909
10	300	6,972,890	10,721,385	3,748,495	2,789,232
Total		69,681,804	86,700,267	17,018,463	12,696,564

HHD: home hemodialysis; ICHD: in-center hemodialysis. Incremental savings equal ICHD expenditure minus HHD expenditure; negative values indicate additional HHD expenditure.

**Table 5:** Present value, NPV and fiscal return indicators under alternative discount rates.

Discount Rate	PV HHD (EUR)	PV ICHD (EUR)	NPV (EUR)	Fiscal BCR	Fiscal ROI
0%	69,681,804	86,700,267	17,018,463	3.44	243.9%
1.5%	64,255,840	78,968,275	14,712,435	3.15	215.1%
3%	59,428,165	72,124,728	12,696,564	2.89	189.3%
5%	53,787,141	64,178,523	10,391,382	2.59	159%

BCR: benefit–cost ratio; HHD: home hemodialysis; ICHD: in-center hemodialysis; NPV: net present value; PV: present value; ROI: return on investment. BCR and ROI are fiscal indicators based only on public-payer cash flows.

## Data Availability

The original contributions presented in this study are included in the article. Further inquiries can be directed to the corresponding author.

## Abbreviations

The following abbreviations are used in this manuscript:

BCR	Benefit–Cost Ratio
CHEERS	Consolidated Health Economic Evaluation Reporting Standards
EOPYY	National Organization for the Provision of Health Services
HHD	Home Hemodialysis
ICHD	In-Center Hemodialysis
ISPOR	International Society for Pharmacoeconomics and Outcomes Research
NPV	Net Present Value
PD	Peritoneal Dialysis
PV	Present Value
QALY	Quality-Adjusted Life-Year
ROI	Return on Investment

## Appendix A

### Appendix A.1. Contextual PD and ICHD Public-Payer Cost Streams

PD is shown only as a public-payer budget benchmark for the same projected patient equivalents. The table does not model a PD program investment pathway and does not imply clinical interchangeability between modalities.

**Table A1:** Contextual annual PD and ICHD public-payer cost streams at a 3% discount rate (EUR).

Year	Patients	PD Cost	ICHD Cost	Incremental PD Expenditure	Discounted Incremental PD Expenditure
1	53	3,368,526	1,894,111	1,474,415	1,431,471
2	142	9,025,108	5,074,789	3,950,319	3,723,555
3	204	12,965,648	7,290,542	5,675,107	5,193,526
4	248	15,762,161	8,863,012	6,899,149	6,129,805
5	279	17,732,431	9,970,888	7,761,543	6,695,175
6	300	19,067,130	10,721,385	8,345,745	6,989,430
7	300	19,067,130	10,721,385	8,345,745	6,785,854
8	300	19,067,130	10,721,385	8,345,745	6,588,208
9	300	19,067,130	10,721,385	8,345,745	6,396,319
10	300	19,067,130	10,721,385	8,345,745	6,210,018
Total		154,189,525	86,700,267	67,489,258	56,143,361

ICHD: in-center hemodialysis; PD: peritoneal dialysis. Incremental PD expenditure equals PD expenditure minus ICHD expenditure.

### Appendix A.2. Deterministic One-Way Sensitivity Analyses

**Table A2:** One-way sensitivity analysis of 10-year NPV at a 3% discount rate.

Parameter	Lower/Alternative Value	NPV (EUR)	Upper/Alternative Value	NPV (EUR)
Annual ICHD comparator cost	EUR 33,085.43 (conventional HD mean)	7,343,367	EUR 41,093.26 (HDF mean)	23,504,410
Annual attrition/non-retention	20%	14,894,750	40%	3,126,623
National training throughput	100 patients/year	1,927,361	200 patients/year	15,016,230
Dedicated medical/nursing staff cost	EUR 520,000/year (−20%)	13,805,490	EUR 780,000/year (+20%)	11,587,637
Machine/water-system acquisition cost	EUR 28,000/set (−20%)	14,869,166	EUR 42,000/set (+20%)	10,523,961
Technical-support cost	EUR 1920/set-year (−20%)	13,589,058	EUR 2880/set-year (+20%)	11,804,069
Home preparation and household equipment	EUR 3680/installation (−20%)	13,520,581	EUR 5520/installation (+20%)	11,872,546
Direct patient-level HHD public cost	EUR 23,765.04/patient-year (−20%)	24,686,948	EUR 35,647.56/patient-year (+20%)	706,179
Staff-training seminar cost	EUR 12,500 in years 1 and 3 (−50%)	12,720,139	EUR 37,500 in years 1 and 3 (+50%)	12,672,988

Base-case NPV: EUR 12,696,564. Each analysis varied one parameter while all other inputs remained at base-case values. Tested values are scenarios, not confidence limits.

### Appendix A.3. Program-Scale Scenarios

**Table A3:** Program-scale scenarios at a 3% discount rate.

Scenario	Target Patients	Annual Throughput	NPV (EUR)	Fiscal BCR	Payback Year
Smaller scale	240	120	8,596,628	2.50	7
Base case	300	150	12,696,564	2.89	6
Larger scale	360	180	16,741,778	3.17	6

The ten-site network and base-case staffing and facilities budgets were held constant. Equipment capacity and procurement and annual training throughput were adjusted proportionally to the target population.
